# Clinical characteristics of Guillain–Barre syndrome in COVID-19: a systematic review and meta-analysis of observational studies

**DOI:** 10.1186/s41983-023-00633-7

**Published:** 2023-03-27

**Authors:** I Gusti Agung Ayu Andra Yusari, Putu Gede Sudira, Dewa Putu Gde Purwa Samatra

**Affiliations:** 1 Faculty of Medicine, Universitas Udayana, Bali, Indonesia; 2 Department of Neurology, Universitas Udayana Hospital, Bali, Indonesia; 3 Department of Neurology, Faculty of Medicine, Universitas Udayana/Prof. Dr. I.G.N.G. Ngoerah General Hospital, Bali, Indonesia

**Keywords:** Guillain–Barre syndrome, COVID-19, Clinical manifestation

## Abstract

**Background:**

Guillain–Barre syndrome (GBS) is a complication that occurs in patients with Coronavirus Disease (COVID-19) infection. The spectrum of symptoms varies from mild to severe symptoms, even death. The study aimed to compare the clinical manifestations in GBS patients with and without COVID-19 comorbidity.

**Results:**

A systematic review and meta-analysis of cohort and cross-sectional studies was conducted comparing the characteristics and course of GBS disease in the COVID-19 positive and COVID-19 negative groups. Four articles were selected with a total sample of 61 COVID-19 positive and 110 COVID-19 negative GBS patients. Based on clinical manifestations, COVID-19 infection increased the likelihood of tetraparesis (OR: 2.54; 95% CI 1.12–5.74; *p* = 0.03) and the presence of facial nerve involvement (OR: 2.34; 95% CI 1.00–5.47; *p* = 0.05). Demyelinating type GBS or AIDP was more common in the COVID-19 positive group (OR: 2.32; 95% CI 1.16–4.61; *p* = 0.02). COVID-19 in GBS significantly increased the need for intensive care (OR: 3.32; 95% CI 1.48–7.46; *p* = 0.004) and the use of mechanical ventilation (OR: 2.42; 95% CI 1.00–5.86; *p* = 0.05).

**Conclusions:**

GBS following COVID-19 infection showed more severe variations in clinical characteristics compared to the group of GBS patients without COVID-19. Early detection of GBS, especially the typical manifestations post COVID-19 infection, is very important to carry out intensive monitoring and early management before the patient's condition worsens.

## Background

Since early 2020, the World Health Organization (WHO) has designated severe acute respiratory syndrome coronavirus 2 (SARS-CoV-2) or coronavirus disease (COVID-19) infection as a pandemic. To date, up to 585 million confirmed cases of COVID-19 have been found, with 6.4 million deaths globally [1].

COVID-19 is known to have respiratory symptoms. Based on the development of research, many manifestations in other organ systems were found, one of which is the nervous system, both the central and peripheral nervous systems. The SARS-CoV-2 receptor, Angiotensin Converting Enzyme (ACE)-2, is also expressed in neural tissue. Nervous system involvement associated with COVID-19 is common, particularly in the form of headache, anosmia, dysgeusia, nausea and vomiting, dizziness, and impaired consciousness. Neurological complications found can be in the form of stroke, seizures, meningitis, encephalitis, and muscle involvement [2, 3].

Guillain–Barre syndrome (GBS) is a form of nervous system involvement in COVID-19, where peripheral nerve damage has occurred [4]. GBS is an inflammatory polyradiculoneuropathy condition characterized by rapid and progressive weakness and is often preceded by an infection. The clinical features, electrophysiological variants, and the course of GBS are diverse and can be influenced by the type of infection that preceded it [5, 6]. The condition in GBS can be life-threatening, because its complications can lead to respiratory system failure that requires intubation and ventilation, bulbar dysfunction, aspiration, and autonomic dysfunction which eventually leads to hemodynamic instability [7].

Two-thirds of the total GBS cases were correlated with the presence of a previous infection. The GBS phenomenon is often associated with epidemics in the world, such as the Zika virus and Middle East respiratory syndrome (MERS) virus epidemics, as well as other viral or bacterial infections [5, 6]. There are reports and case series from various countries that reported cases of GBS associated with COVID-19 [8]. It is estimated that the prevalence of GBS cases is 15 cases in every 100,000 cases of COVID-19 infection [9].

Several systematic reviews and meta-analyses of GBS cases in COVID-19 have also been carried out in previous studies. However, there have been no systematic reviews or meta-analyses comparing the clinical characteristics and outcomes of GBS patients infected with COVID-19 and GBS without previous COVID-19 infection, especially from observational studies.

Therefore, this systematic review and meta-analysis aims to describe the clinical characteristics of GBS associated with COVID-19 from observational studies, and to compare them with the group of GBS not associated with COVID-19 infection. By knowing the comparison between the two groups, it is hoped that it can help health practitioners understand the typical clinical manifestations of GBS with COVID-19, and be able to establish an initial diagnosis accompanied by appropriate management.

## Methods

**Fig. 1:**
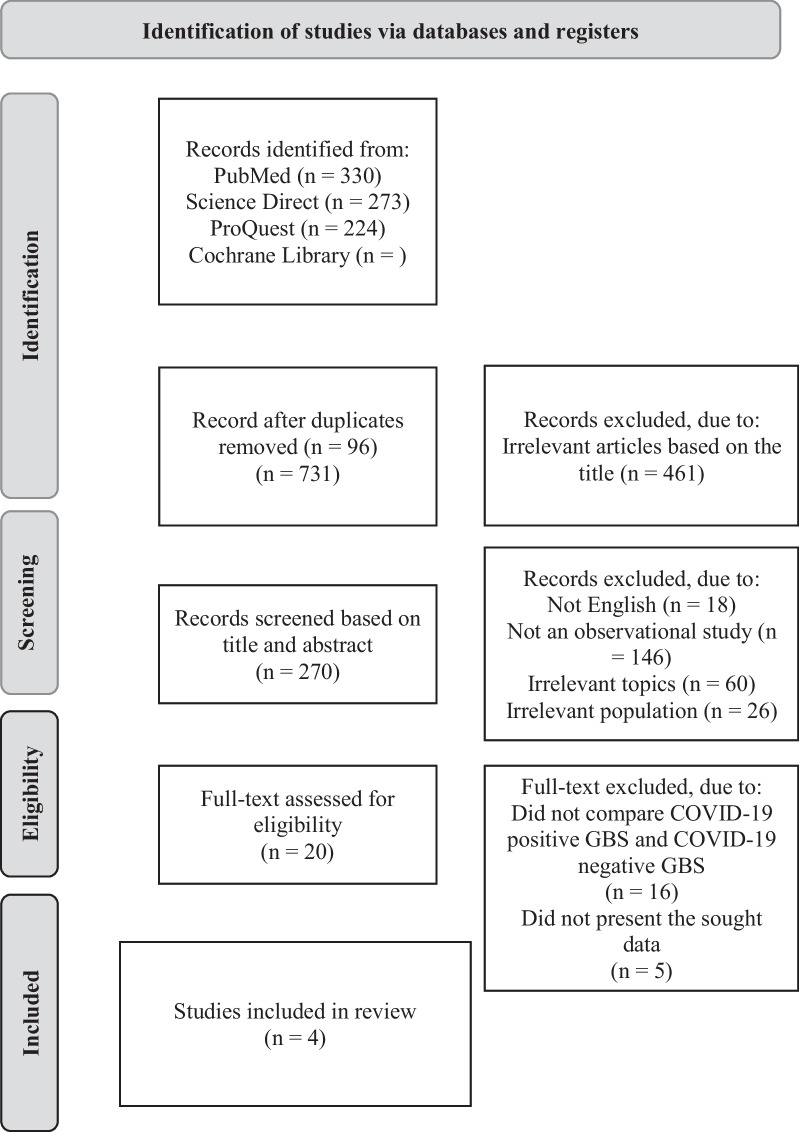
Article selection flow based on PRISMA method

A systematic literature search was conducted in the PubMed, Proquest, and Science Direct databases using the keywords “Guillain–Barre syndrome” combined with “COVID-19”, “SARS-CoV-2”, and “coronavirus” published in the year of 2019 until the first half of July 2022. Article selection was carried out using the Preferred Reporting Items for Systematic Reviews and Meta-Analyses (PRISMA) method (Fig. 1). Search results from all three databases were combined and duplicate articles were excluded. Articles were then selected based on the title and suitability of the topic sought, then more specifically selected based on the title and abstract to assess the type and method of study as well as the population being studied. Finally, the articles were screened based on the full-text manuscript and selected for the analysis.

Inclusion criteria were observational research articles with cohort, case–control, or cross-sectional study methods, which presented characteristic data from groups of GBS patients with and without COVID-19 infection and were written in English. The excluded literature is literature reviews, systematic reviews, case reports, abstracts, proceedings, editorials, or research that does not display the data sought in the inclusion criteria. Several articles were evaluated for their entire manuscript to assess study eligibility. The quality of the selected studies was assessed using the Newcastle–Ottawa Scale (NOS) based on the selection criteria, comparability, and outcomes of the study (Table 1). The risk of bias is not systematically assessed.

**Table 1:** Assessment of study quality based on the Newcastle–Ottawa Scale (NOS)

Study (cohort)	Selection				Comparability	Outcome			Total score
	Representative of the exposed cohort	Selection of non-exposed cohort	Ascertainment of exposure	Demonstration that outcome of interest was not present at start of study	Comparability of cohorts on the basis of the design or analysis	Assessment of outcome	Was follow-up long enough for outcomes to occur	Adequacy of follow-up of cohorts	
Filosto, 2020	1	1	1	1	2	1	0	1	8
Keddie, 2021	1	1	1	1	2	1	1	1	9
Luijten, 2021	1	1	1	1	2	1	1	0	8

Study (cross-sectional)	Selection				Comparability	Outcome		Total Score
	Representativeness of the sample	Sample size	Non-respondents	Ascertainment of exposure (risk factor)	The subjects in different outcome groups are comparable, based on the study design or analysis. Confounding factors are controlled	Assessment of outcome	Statistical test	
Lopez-Hernandez, 2021	1	0	1	2	1	1	1	7

Data were collected from each study and extracted based on the time of publication, country of study, study method or design, number of study participants, as well as summary of the findings of the variables in each study. The research variables extracted and analyzed were demographic data in the form of gender and age as well as patient comorbidities. Clinical characteristics were assessed based on clinical neurologic and supporting examinations. The clinical neurologic evaluation assessed were weakness, GBS disability score, muscle strength score, cranial nerve involvement, and presence of autonomic dysfunction. Supporting examinations were electrophysiological features that showed variant GBS and the presence of cytoalbuminologic dissociation in the cerebrospinal fluid. Patient outcomes were assessed based on the type of care required, such as intensive care, the need for mechanical ventilation, and the presence of mortality. Data was also collected by combining these variables in both groups of GBS patients from the selected studies.

Data analysis was performed using Review Manager software version 5.4.1. Each variable was grouped and compared between the GBS group with and without COVID-19 infection. Variables can be dichotomous data that is recorded based on the number of events between groups or continuous data that is inputted based on the mean and standard deviation. The results of the analysis were calculated with 95% Confidence Interval (CI) and forest plots. Random-effect analysis was performed if heterogeneity based on *I*^2^ was more than 50%, and fixed-effect analysis was performed if the *I*^2^ was less than 50%.

## Results

Through the article selection process in Fig. 1, four observational research articles were included in this study. One study was a retrospective cohort study [10], two studies were prospective cohort studies [5, 6], and one study is a cross-sectional study [11]. The studies were carried out in countries in Europe (Italy, Switzerland, Netherlands, UK), Asia (China), and South America (Mexico). The study quality assessment based on NOS showed one study with a score of 9, two studies with a score of 8, and one study with a score of 7 out of a maximum score of 9. Therefore, the studies included in this analysis were of good quality.

There were 171 total GBS samples in this study, with 61 samples from the GBS group positive for COVID-19 and 110 samples from the GBS group negative for COVID-19. Demographic data of patients were the mean of age and gender. Patients’ comorbidities were also obtained in both groups. Specifically for the COVID-19 positive group in the cohort study, data on the average interval of days from being infected with COVID-19 before the first manifestation of GBS were shown (Appendix 1).

Regarding the clinical characteristics of GBS, there were manifestations of weakness in the form of tetraparesis or paraparesis as well as a GBS disability score (Hughes Functional Grading Scale) and a muscle strength score based on Medical Research Council (MRC). Parameters of cranial nerve involvement are seen generally and specifically on the oculomotor nerve, facial nerve, or bulbar nerve. The presence of autonomic dysfunction is also included. Electrophysiology examinations were included to determine the variant of GBS, and examination of cerebrospinal fluid to see the presence of cytoalbuminologic dissociation. The patient's clinical outcome was assessed based on the need for intensive care in the intensive care unit (ICU) and the use of a mechanical ventilator. Two studies showed mortality data.

Descriptive data of the clinical characteristics are described in Table 2. While a summary of findings of the variables from each study is described in Appendix 1. Table 2 combines the data from each study and calculates the percentage to show the differences between the two groups descriptively. Not all studies included the same variables, where there were some variables which were only shown by two or three studies. Appendix 1 shows which studies examined the related variables, and which studies did not evaluate those variables.

**Table 2:** Clinical characteristics of COVID-19 positive and COVID-19 negative GBS patients from four selected studies

Characteristic	COVID-19 positive GBS		COVID-19 negative GBS	
	*n* (61)	%	*n* (110)	%
Gender				
Male	44	72.1	68	61.8
Female	17	27.9	42	38.2
Mean of age	56.4 ± 8.64		52.3 ± 5.37	
Comorbidity				
Obesity	7 (*n* = 37)	18.9	16 (*n* = 52)	30.8
Diabetes	7 (*n* = 50)	14.0	7 (*n* = 74)	9.5
Hypertension	19 (*n* = 50)	38.0	12 (*n* = 74)	16.2
Weakness				
Tetraparesis	42 (*n* = 54)	77.8	43 (*n* = 75)	57.3
Paraparesis	3 (*n* = 54)	5.6	15 (*n* = 75)	20.0
Mean of MRC score	36.7 ± 14.48		42.3 ± 9.27	
GBS disability score ≥ 3	25 (*n* = 31)	80.6	64 (*n* = 91)	70.3
Cranial nerve involvement^a^	42	68.9	44	40.0
Oculomotor nerve	4 (*n* = 48)	8.3	16 (*n* = 88)	18.1
Facial nerve	22 (*n* = 48)	45.8	29 (*n* = 88)	32.9
Bulbar nerve	14 (*n* = 48)	29.1	26 (*n* = 88)	29.5
Autonomic dysfunction	33 (*n* = 48)	68.7	20 (*n* = 86)	23.2
GBS variant and neurophysiology				
AIDP	40	65.6	44	40.0
AMAN/AMSAN	4	6.6	25	22.7
Miller fisher	3	4.9	6	5.5
Equivocal	5	8.2	17	15.5
Normal neurophysiology	0	0	2	1.8
Neurophysiology not assessed	9	14.7	16	14.5
Cerebrospinal fluid				
Cytoalbuminologic dissociation	19 (*n* = 48)	39.6	49 (*n* = 88)	55.7
Outcome				
Intensive care unit	28 (*n* = 54)	51.9	19 (*n* = 75)	25.3
Mechanical ventilation	13 (*n* = 31)	41.9	21 (*n* = 91)	23.1
Mortality	3 (*n* = 24)	12.5	1 (*n* = 56)	1.8

*GBS* Guillain–Barre syndrome, *COVID-19* Coronavirus Disease 2019, *MRC* Medical Research Council grading system for evaluation of muscle strength, *AIDP* acute inflammatory demyelinating polyneuropathy, *AMAN* acute motor axonal neuropathy, *AMSAN* acute motor-sensory axonal neuropathy

*n*: If the entire population in the study includes these variables, the value of *n* corresponds to the total number of samples in each group. Therefore, the value of *n* varies depending on the number of populations included in the analysis of these variables

^a^One patient could have more than one cranial nerve involvements

A meta-analysis was conducted on each variable to analyze whether COVID-19 infection affected the clinical picture of patients with GBS. The results of the analysis presented can be seen in Table 3. The forest plot test for each variable can be seen in Appendix 2.

**Table 3:** Meta-analysis of clinical characteristics of GBS in COVID-19

Variable	Number of studies	Participants	Statistic method	Estimated effect (95% CI)	*I*^2^ (%)	*p* value
Demographic data						
Age	4	171	Mean difference (IV, fixed, 95% CI)	6.47 [1.34, 11.61]	38	0.01*
Male	4	171	Odds ratio (M–H, fixed, 95% CI)	1.64 [0.80, 3.38]	0	0.18
Female	4	171	Odds ratio (M–H, fixed, 95% CI)	0.61 [0.30, 1.25]	0	0.18
Comorbidity						
Obesity	2	89	Odds ratio (M–H, fixed, 95% CI)	0.59 [0.18, 1.88]	0	0.37
Diabetes	3	124	Odds ratio (M–H, fixed, 95% CI)	1.03 [0.32, 3.31]	0	0.96
Hypertension	3	124	Odds ratio (M–H, fixed, 95% CI)	1.75 [0.69, 4.41]	0	0.24
Clinical neurologic manifestation						
Tetraparesis	3	129	Odds ratio (M–H, fixed, 95% CI)	2.54 [1.12, 5.74]	2	0.03*
Paraparesis	3	129	Odds ratio (M–H, fixed, 95% CI)	0.23 [0.07, 0.76]	37	0.02*
MRC score	3	136	Mean difference (IV, random, 95% CI)	− 5.97 [− 16.52, 4.58]	62	0.27
GBS disability score ≥ 3	3	122	Odds ratio (M–H, fixed, 95% CI)	1.92 [0.68, 5.39]	0	0.22
Cranial nerve involvement	4	171	Odds ratio (M–H, random, 95% CI)	2.54 [0.43, 14.91]	75	0.3
Oculomotor	3	136	Odds ratio (M–H, fixed, 95% CI)	0.58 [0.17, 2.06]	0	0.4
Facial	3	136	Odds ratio (M–H, fixed, 95% CI)	2.34 [1.00, 5.47]	0	0.05*
Bulbar	3	136	Odds ratio (M–H, fixed, 95% CI)	2.03 [0.76, 5.41]	0	0.16
Autonomic Dysfunction	3	134	Odds ratio (M–H, random, 95% CI)	4.26 [0.63, 28.57]	75	0.14
GBS variant						
AIDP	4	171	Odds ratio (M–H, fixed, 95% CI)	2.32 [1.16, 4.61]	47	0.02*
AMAN/AMSAN	4	171	Odds ratio (M–H, fixed, 95% CI)	0.27 [0.09, 0.76]	17	0.01*
Miller–Fisher	4	171	Odds ratio (M–H, fixed, 95% CI)	2.06 [0.53, 7.96]	0	0.29
Cerebrospinal fluid						
Cytoalbuminologic dissociation	3	136	Odds ratio (M–H, fixed, 95% CI)	0.91 [0.39, 2.07]	0	0.81
Outcome						
Intensive care unit	3	129	Odds ratio (M–H, fixed, 95% CI)	3.32 [1.48, 7.46]	0	0.004*
Mechanical ventilation	3	122	Odds ratio (M–H, fixed, 95% CI)	2.42 [1.00, 5.86]	0	0.05*
Mortality	2	80	Odds ratio (M–H, fixed, 95% CI)	6.45 [0.86, 48.22]	0	0.07

*M–H* Mantel–Haenszel test which provides a pooled odds ratio, *95% CI* 95% confidence interval, *GBS* Guillain–Barre syndrome, *MRC* Medical Research Council grading system for evaluation of muscle strength, *AIDP* acute inflammatory demyelinating polyneuropathy, *AMAN* acute motor axonal neuropathy, *AMSAN* acute motor-sensory axonal neuropathy

*I*^2^: Heterogeneity

*p* value: effect size (value ≤ 0.05 is significant; [*]: significant value)

In demographic data, it was found that the GBS group with COVID-19 tended to be older than the GBS group without COVID-19 (MD: 6.47; 95% CI 1.34–11.61; *p* = 0.01). Meanwhile, based on comorbidity, there was no difference between the case group and the control group.

The manifestation of GBS weakness in COVID-19 tended to show the tetraparesis type (OR: 2.54; 95% CI 1.12–5.74; *p* = 0.03). Whereas in GBS without COVID-19, it significantly showed paraparesis type of weakness (OR: 0.23; 95% CI 0.07–0.76; *p* = 0.02). There were no significant differences in MRC scores and high disability scores between the two groups. In the analysis of cranial nerve involvement, the only significant difference was found in the manifestation of facial nerve involvement which tended to be more common in GBS with COVID-19 (OR: 2.34; 95% CI 1.00–5.47; *p* = 0.05). The incidence of autonomic dysfunction was the same in both groups.

The GBS variant based on electrophysiological examination also showed significant analysis results. GBS in COVID-19 tended to have a variant type of acute inflammatory demyelinating polyneuropathy or AIDP (OR: 2.32; 95% CI 1.16–4.61; *p* = 0.02). Meanwhile, axonal GBS such as acute motor axonal neuropathy (AMAN) and acute motor sensory axonal neuropathy (AMSAN) tended to occur more frequently in GBS without COVID-19 (OR: 0.27; 95% CI 0.09–0.76; *p* = 0.01).

Outcome of GBS cases in COVID-19 was assessed based on the treatment the patient underwent. COVID-19 in GBS significantly increased the need for intensive care (OR: 3.32; 95% CI 1.48–7.46; *p* = 0.004), and more likely to require the use of mechanical ventilation (OR: 2.42; 95% CI 1.00–5.86; *p* = 0.05). Mortality between the two groups were the same, but this cannot be confirmed, because studies examining GBS mortality in COVID-19 and the number of participants were smaller.

## Discussion

GBS is the most common neuromuscular complication of COVID-19 infection [12]. In some studies, the prevalence of GBS has increased during the COVID-19 pandemic. In a study in Italy, it was found that the incidence of GBS increased by 2.6 times during the pandemic period of March–April 2020 compared to the period of March–April 2019, where 88% of GBS cases were diagnosed as positive for COVID-19 [10]. Study in Spain also showed an increased incidence of GBS in COVID-19 compared to patients without COVID-19 [13]. Based on the analysis of several case reports, it was found that GBS in COVID-19 was most commonly found in high and middle to upper income countries such as in Europe. The incidence of GBS in COVID-19 in low-to-lower-middle income countries is underreported. This is allegedly due to the low initiative of the community to seek health facilities when sick, especially during the pandemic, due to social problems or stigma, facilities, and the economy [12].

There is no certainty regarding the pathophysiological mechanism of GBS in COVID-19. The hypothesis of GBS in COVID-19 is suspected to occur through the post-infection inflammatory process. COVID-19 enhances the immune response facilitated by T cell activation accompanied by an increase in inflammatory markers and pro-inflammatory cytokines [14]. In addition, the pathophysiology of GBS in COVID-19 can be grouped based on the onset of COVID-19 infection itself, namely, para-infectious and post-infectious mechanisms. The para-infectious mechanism occurs in the acute phase of COVID-19 infection through immune dysregulation and cytokine release that causes direct damage to nerve roots and cells [8, 12]. Whereas in the post-infectious mechanism, there is a molecular mimicry mechanism in which autoantibodies produced by the immune response to the SARS-CoV-2 pathogen reacted with similar peripheral nerve structures, causing damage to these peripheral nerves [14, 15]. However, determining the pathophysiology based on the onset between COVID-19 infection and the occurrence of GBS is considered inappropriate, because the symptoms of COVID-19 do not always appear in a uniform time and can have a longer incubation period [8].

In previous reports and studies, most of the patients were over 50 years and dominated by men. Older age and male gender are associated with more severe COVID-19 conditions [14]. In this study, the average age in both GBS groups was above 50 years, although statistically the COVID-19 positive group tended to be older. Patients with GBS were mostly male in both groups of this study.

GBS has a variety of clinical presentations, which are generally characterized by bilateral flaccid weakness with a course of weakness from the lower extremities upwards or ascending paralysis [16]. This study found tetraparesis is a feature of weakness that is often found in GBS with COVID-19. Meanwhile, paraparesis tends to occur more in the GBS group without COVID-19. This is supported by a study on the topography of GBS in India before the pandemic, where paraparesis was the most common feature of GBS (60%), and there were no cases with progression to tetraparesis [17].

Tetraparesis is associated with a more severe GBS outcome, with more frequent cranial nerve involvement and more severe lower extremity weakness than paraparesis. In a cohort study, 98% of patients with paraparesis were significantly more recoverable and able to walk unaided than patients with tetraparesis [18]. This is in accordance with the analysis of this study, where GBS patients with COVID-19 have a more severe condition that requires intensive care and the use of mechanical ventilation.

The mechanism of autoimmune neuropathy in COVID-19 is associated with cranial nerve weakness [9]. The supporting clinical picture is that one of the cranial nerves, namely, the facial nerve, was found to be significantly more frequent in the COVID-19 group. Autoimmune neuropathy can cause bulbar nerve involvement, such as the glossopharyngeal, vagus, and hypoglossal nerves, although in this study there was no significant difference in bulbar nerve involvement between the two groups. Involvement of the nerves of the respiratory muscles due to the presence of this autoimmune neuropathy cause patient's deterioration to the point of requiring mechanical ventilation. Involvement of the autonomic nervous system also contributes to increased patient morbidity and mortality, because it can lead to cardiac arrhythmias and blood pressure instability. Therefore, monitoring in the intensive care unit is very necessary [19].

This is consistent with the data in Table 2, where the GBS group with COVID-19 had a higher percentage of autonomic dysfunction (68.7%) than without COVID-19 (23.2%), although it was not significant in the forest plot analysis. Therefore, in the end, more patients in the GBS group with COVID-19 required intensive care and the use of mechanical ventilators. In two studies, mortality data was even found in GBS patients with previous COVID-19 infection.

Examinations which help establishing the diagnosis of GBS are electrophysiological and cerebrospinal fluid examinations. The demyelinating variant or AIDP is mostly found in GBS with COVID-19. AIDP is associated with the infiltration mechanism of T lymphocytes and macrophages that erode the myelin structure. Autoimmune processes and the formation of antibodies against myelin glycolipids lead to demyelination [20]. Axonal type GBS tended to be more common in GBS studies without COVID-19, which is 59% compared to the myelinated type (33%) [17]. Axonal GBS is often associated with previous Campylobacter jejuni infection [12]. This is in accordance with the results of the analysis, where axonal GBS such as AMAN and AMSAN significantly tend to occur in the GBS group without COVID-19.

Examination of the cerebrospinal fluid in GBS usually shows an increase in protein with normal white blood cells, otherwise known as cytoalbuminologic dissociation. Previous studies have not found the presence of the SARS-CoV-2 virus in the cerebrospinal fluid [21]. So that the comparative analysis related to the examination of cerebrospinal fluid between the two groups did not have a significant difference.

Early detection of GBS, especially the typical manifestations of post COVID-19 infection, is very important in efforts to carry out intensive monitoring and early management before the patient's condition worsens. Giving therapy such as immunotherapy is said to have a better response in the first 2 weeks, especially in patients with GBS disability scores ≥ 3 [22]. Therefore, this study helps to describe the typical clinical manifestations of GBS with COVID-19 to improve an optimal diagnosis and appropriate management.

## Conclusion

COVID-19 infection that preceded GBS showed a more severe variation of clinical manifestations than the group of GBS patients without COVID-19. The limitation of this study is that there were very few observational studies regarding the comparison of GBS patients with and without previous COVID-19 infection. Outcomes showing the mortality rate and prognosis of GBS patients in COVID-19 are also limited; therefore, further analysis is needed to obtain results that are more representative of conditions in the field.

## Data Availability

The data used and analyzed in this study are available from the corresponding author on reasonable request.

## Appendix 1: Summary of findings in four selected studies regarding comparison of characteristics of GBS patients in the COVID-19 positive and COVID-19 negative groups

**Table:**

Main author, years	Types of study	Country	Participants	Interval from COVID-19 symptoms to GBS symptoms	Gender	Mean of age	Comorbidity
Filosto, 2020	Retrospective Cohort	Italy	COVID-19 Positive: 30 patients COVID-19 Negative: 17 patients	23 days (IQR: 16–35 days)	Male Female	COVID-19 Positive: 59,2 years COVID-19 Negative: 57.2 years	Obesity Diabetes Hypertension
Keddie, 2021	Prospective Cohort	United Kingdom	COVID-19 Positive: 13 patients COVID-19 Negative: 22 patients	12 days (IQR: 4–21 days)	Male Female	COVID-19 Positive: 60 years COVID-19 Negative: 54.5 years	Diabetes Hypertension
Luijten, 2021	Prospective Cohort	Chinese, Italian, Swiss, Netherland	COVID-19 Positive: 11 patients COVID-19 Negative: 36 patients	16 days (IQR: 12–22 days)	Male Female	COVID-19 Positive: 63 years COVID-19 Negative: 53 years	Not assessed
Lopez-Hernandez, 2021	Cross-sectional	Mexico	COVID-19 Positive: 7 patients COVID-19 Negative: 35 patients	Not assessed	Male Female	COVID-19 Positive: 43,4 years COVID-19 Negative: 44.5 years	Obesity Diabetes Hypertension

Main author, years	Weakness	Mean of MRC score	GBS disability score ≥ 3	Cranial nerve involvement	Autonomic dysfunction	GBS variant and neurophysiology	Cerebrospinal fluid	Outcome
Clinical characteristics								
Filosto, 2020	Tetraparesis Paraparesis	COVID-19 Positive: 26,3 COVID-19 Negative: 41.4	Not assessed	General Oculomotor Facial Bulbar	COVID-19 Positive: 27 patients COVID-19 Negative: 4 patients	AIDP AMAN/AMSAN Equivocal Normal	Cytoalbuminologic Dissociation	ICU
Keddie, 2021	Tetraparesis Paraparesis	Not assessed	COVID-19 Positive: 61,5% COVID-19 Negative: 63.6%	General	Not assessed	AIDP AMAN/AMSAN Miller Fisher Normal	Not assessed	ICU Mechanical Ventilation Mortality
Luijten, 2021	Tetraparesis Paraparesis	COVID-19 Positive: 51 COVID-19 Negative: 51	COVID-19 Positive: 100% COVID-19 Negative: 76.4%	General Oculomotor Facial Bulbar	COVID-19 Positive: 4 patients COVID-19 Negative: 7 patients	AIDP AMAN/AMSAN Miller Fisher Equivocal	Cytoalbuminologic Dissociation	ICU Mechanical Ventilation Mortality
Lopez-Hernandez, 2021	Not assessed	COVID-19 Positive: 32,8 COVID-19 Negative: 34.7	COVID-19 Positive: 85,7% COVID-19 Negative: 68.5%	General Oculomotor Facial Bulbar	COVID-19 Positive: 2 patients COVID-19 Negative: 9 patients	AIDP AMAN/AMSAN Miller Fisher Equivocal	Cytoalbuminologic Dissociation	Mechanical Ventilation

## Abbreviations

ACE: Angiotensin converting enzyme
AIDP: Acute inflammatory demyelinating polyneuropathy
AMAN: Acute motor axonal neuropathy
AMSAN: Acute motor-sensory axonal neuropathy
CI: Confidence interval
COVID-19: Coronavirus disease 2019
GBS: Guillain–Barre syndrome
ICU: Intensive care unit
MD: Mean difference
MERS: Middle east respiratory syndrome
MRC: Medical Research Council
NOS: Newcastle–Ottawa Scale
OR: Odds ratio
PRISMA: Preferred reporting items for systematic reviews and meta-analyse
SARS-CoV-2: Severe acute respiratory syndrome coronavirus 2
WHO: World Health Organization
